# Artificial Neural Network Approach to Guarantee the Positioning Accuracy of Moving Robots by Using the Integration of IMU/UWB with Motion Capture System Data Fusion

**DOI:** 10.3390/s22155737

**Published:** 2022-07-31

**Authors:** Ahmed M. M. Almassri, Natsuki Shirasawa, Amarbold Purev, Kaito Uehara, Wataru Oshiumi, Satoru Mishima, Hiroaki Wagatsuma

**Affiliations:** 1 Graduate School of Life Science and Systems Engineering, Kyushu Institute of Technology (Kyutech), 2-4 Hibikino, Wakamatsu-ku, Kitakyushu 808-0196, Japan; natsuki.shirasawa189@mail.kyutech.jp (N.S.); purev.amarbold118@mail.kyutech.jp (A.P.); uehara.kaito505@mail.kyutech.jp (K.U.); oshiumi.wataru745@mail.kyutech.jp (W.O.); mishima.satoru151@mail.kyutech.jp (S.M.); waga@brain.kyutech.ac.jp (H.W.); 2 Robotic Innovation Research Center (RIRC), Israa University, Gaza P860, Palestine

**Keywords:** ultra-wideband, inertial measurement units, motion capture system, sensor fusion, neural network, indoor positioning system, robot position measurement

## Abstract

This study presents an effective artificial neural network (ANN) approach to combine measurements from inertial measurement units (IMUs) and time-of-flight (TOF) measurements from an ultra-wideband (UWB) system with OptiTrack Motion Capture System (OptiT-MCS) data to guarantee the positioning accuracy of motion tracking in indoor environments. The proposed fusion approach unifies the following advantages of both technologies: high data rates from the MCS, and global translational precision from the inertial measurement unit (IMU)/UWB localization system. Consequently, it leads to accurate position estimates when compared with data from the IMU/UWB system relative to the OptiT-MCS reference system. The calibrations of the positioning IMU/UWB and MCS systems are utilized in real-time movement with a diverse set of motion recordings using a mobile robot. The proposed neural network (NN) approach experimentally revealed accurate position estimates, giving an enhancement average mean absolute percentage error (MAPE) of 17.56% and 7.48% in the X and Y coordinates, respectively, and the coefficient of correlation R greater than 99%. Moreover, the experimental results prove that the proposed NN fusion is capable of maintaining high accuracy in position estimates while preventing drift errors from increasing in an unbounded manner, implying that the proposed approach is more effective than the compared approaches.

## 1. Introduction

With recent advances in portable sensing techniques, the use of wearable sensors in indoor environments to monitor the location of a human and performance has been attracting increasing attention, particularly in robotic workplaces. In addition to providing increased safety for human operators, tracking humans also facilitates intelligent human–robot interactions. The development of sensing technologies and sensor signal-processing techniques has paved the way for this purpose. One of the biggest challenges in indoor motion tracking for human–robot interaction is accurate estimation with non-invasive sensors, non-limited workspace, and non-visible regions. Recently, inertial measurement unit (IMU) technology has provided an impetus in motion tracking research in indoor environments, particularly for applications in which motion tracking based on optical technologies is unsuitable [1]. Such technology is ubiquitously used in numerous applications, including healthcare monitoring, intelligent environments [2], lower-limb prosthetic tracking, assisted navigation [3], human movement analysis [4], and human-robot interaction [5]. Moreover, the use of IMU technology has received enormous attention owing to its various advantages, including being small, lightweight, and low-cost. Typically, IMUs are composed of accelerometers and gyroscopes and can be combined with other sensors, such as ultrasound, barometers, and magnetometers [6].

IMUs do not suffer from occlusions and are considerably more affordable and far less intrusive than optical systems and mechanical trackers. However, both accelerometer and gyroscope measurements are still limited from time-varying biases and noise, which leads to significant drift over time, which makes the output of the IMU unreliable after a few seconds [7]. Therefore, various algorithms and hardware have been studied to solve the drift issues. Some solutions exploit technologies such as ultrasonic sensors [8], GPS [9], ultra-wideband (UWB) [10], and cameras [11]. Another known solution involves integrating IMUs with a three-axis magnetometer, in which a local (earth) magnetic field is measured and used as an earth-fixed reference for the instantaneous estimation of the IMU orientation [1].

Following the above trends, a variety of approaches to IMU-based human motion tracking have been investigated. It has also increased over the past few years owing to emerging technologies, such as exoskeletons, vision-based systems, and motion capture based on inertial systems [1]. Researchers have dedicated their knowledge to improving the accuracy of IMU-based human motion tracking data measurements by estimating the systematic errors of the IMU sensors and correcting the positioning errors. Furthermore, sensor fusion techniques have been implemented to derive useful information from IMUs [12,13,14]. The integration of IMUs with UWB sensors is one of the best approaches for achieving high positioning accuracy [15,16,17]. This is where the fused UWB system and IMUs can assist in eliminating noise and enhancing the measurements because they combine the features of different mechanisms to enhance performance [18]. However, sensor fusion IMUs with UWBs are still impeded by limited accuracy, particularly when a large range of different dynamic and static rotational and translational motions are considered [19]. Accordingly, in this study, we present an indoor positioning approach in real-time that fuses the UWB system and IMUs with OptiTrack Motion Capture System (OptiT-MCS) data using an artificial neural network (ANN) to guarantee the positioning accuracy of motion tracking using moving robots.

**Figure 1:**
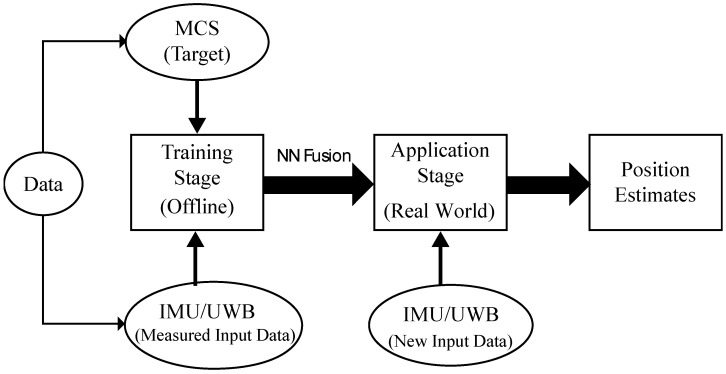
Block diagram of the neural network in training and application stages.

Figure 1 shows the block diagram of the proposed ANN system during the training and application stages. The calibrated data were divided into three subsets including training, validation, and test. In the training stage, the training set used for computing the gradient and updating the network weights and biases as well as the error on the validation set was monitored during the training process. In this stage, the network weights and biases are saved at the minimum of the validation set error. Accordingly, the training stage was performed offline using the integration position data of IMU/UWB and MCS that was pre-recorded from mobile robot motions during experiments. It took a few minutes using 1000 epochs to determine the network weights and biases with a minimum validation error. Then, the test set was only used to explore the performance of the training stage and confirm its generalizability. While in the application stage in a real-world environment, a new independent input data set (IMU/UWB) was used on which the algorithm had never been trained on and the response time was in milliseconds, which does not affect the real-time system performance. The MCS data are only used in the training stage as a target in the output layer of NN and will not be used later in the application stage.

The motivation of the paper is to address the issue of position accuracy in indoor motion tracking with non-visible regions. It is observed that correcting the positioning errors obtained from the integration of IMUs with UWB sensors using existing approaches was still impeded by limited accuracy. Especially in places where human and robots work collaboratively, control systems to ensure the safety and accuracy of movements is highly demanded. In this paper, the proposed ANN fusion approach using IMU/UWB and MCS data overcomes the issue of accuracy and helps to accurately obtain information about the location of moving robots. This led to tracking both human and robot positions and their dynamic movements with high accuracy that contributes to a stronger interaction between human and machine, which will no longer lead to a complete shutdown of the machine, but rather to a reasonable decrease in operating speed or adjustment of movement directions.

The objectives of this study are as follows: (1) to improve the accuracy of positioning and distancing measurements by integrating IMU/UWB with MCS data fusion using a neural network; and (2) to investigate and evaluate the error accumulation across the entire motion tracking process with high-speed data transfer at very low power levels with low-cost time-of-flight (ToF) sensors. The contributions of the paper are listed as follows:

1. The ANN fusion approach is proposed to guarantee the positioning accuracy of moving robots in line-of-sight (LOS) scenarios.
2. Practical OptiT-MCS with mobile robot trajectory position tracking is designed and conducted to verify the proposed approach. The OptiT-MCS is only used for training the NN as a reference (target) for the IMU/UWB system to enrich the dataset with high accuracy and will no longer be used in the application stage. The results reveal that our proposed method is able to achieve high accuracy in position estimates and prevents drift errors from increasing unboundedly compared to existing methods.
3. The ANN fusion approach can replace the vision-based and optical systems in positioning tracking, which leads to more accuracy with less complexity especially in human–robot interaction where area can dynamically change due to operator movements.

The remainder of this paper is organized as follows: In Section 2, we provide more background to the current work by relating it to previous work in the field. Section 3 discusses the method of the measurement system used as well as the system design and integration of IMU/UWB with OptiT-MCS to perform positioning tracking in real time. In addition, data collection and an ANN model are also presented. In Section 4, the results obtained from the proposed system are examined and discussed, and the system is validated and evaluated. Finally, in Section 5, we present our conclusions and suggest future research directions.

## 2. Related Work

Motion and positioning tracking for humans in indoor environments has received the attention and efforts of generations of researchers. The use of wearable sensor-based sensing technologies to track human motion has been increasing. IMUs are often used in this application and have been widely studied in recent years because they are cost-effective technologies that enable human performance assessment and human–robot interaction [1]. In this study, we present an approach that combines measurements from IMUs and TOF measurements from a UWB system with MCS data fusion using an ANN for indoor positioning and distancing with a high level of accuracy. This work was motivated by promising technology using UWB in terms of accuracy for indoor positioning and tracking. The proposed IMU/UWB system consists of a network of asynchronous and stationary (rigidly fixed, mounted) transmitters and receivers. Related surveys and descriptions of UWB technology are summarized by [18] and a more general introduction to UWB technology and its use in positioning applications can be found in [20,21].

UWB technology is capable of precise range measurement and has been accurately applied in numerous indoor positioning systems owing to its high time-domain resolution. Generally, the estimation measurements are implemented using many positioning algorithms that can be classified into the following five main categories: (1) time-of-flight (TOF), (2) angle of arrival (AOA), (3) received signal strength (RSS), (4) time difference of arrival (TDOA), and (5) hybrid algorithm [18]. The most commonly used positioning method uses basic geometry by measuring the transmitter position from the TOF measurements. This method is called trilateration (or multi-lateration, if more than three anchors are used). The drawback of this approach is that the measurements are not perfect because of noise, and the constructed time difference of arrival (TDOA) measurements are no longer independently distributed, which complicates the calculations and causes errors in the estimated position and orientation [17]. Despite its potential, UWB measurements also suffer from the multi-path phenomenon, particularly when there is no clear line-of-sight (LOS) between the transmitter and receiver, resulting in measurement outliers and causing the estimation error to increase up to several meters [22]. Accordingly, the detection of non-line-of-sight (NLOS) conditions and the mitigation of their effects as well as the problem of how to robustly deal with outliers in the measurements of UWB-based systems have received significant attention, e.g., [23,24,25,26]. Several techniques and approaches have been proposed as solutions to UWB system limitations. One method involves modelling the outliers of UWB measurements in terms of the probability of NLOS and introducing a delay, either by a shifted Gaussian or an exponential [27,28]. The drawback of this approach is that the Gaussian distribution is clearly not suitable owing to the large number of outliers, as it excludes the physically unreasonable possibility of pulses travelling faster than the speed of light. A solution to this problem was proposed in [17], which involves modelling UWB measurements using a heavy-tailed asymmetric distribution. Based on [17], the problem is formulated as a maximum posteriori (MAP) problem, which is then solved using an optimization approach. Other approaches use statistical analysis of the resulting range measurements [29,30], whereas another set of algorithms is based on the study of the channel impulse response (CIR) [31,32]. The limitations of this method require some form of additional information, such as a map of the scenario. Besides, the possibility of obtaining the CIR could be limited.

Meanwhile, most researchers have studied human motion-based wearable sensors from different perspectives, either with a focus on the application [33] or technical aspects [34]. For example, a review of wearable sensors for human monitoring using motion capture based on inertial sensors was proposed in [35]. Similarly, in another review paper [36], the authors focused on the applications of wearable sensors to human activity recognition and investigated the limitations and advantages of multi-sensor fusion in body sensor networks. In contrast, the devices and sensors that are used for motion tracking are discussed in [37] and the authors review the applications of wearable sensors to biomechanics.

**Figure 2:**
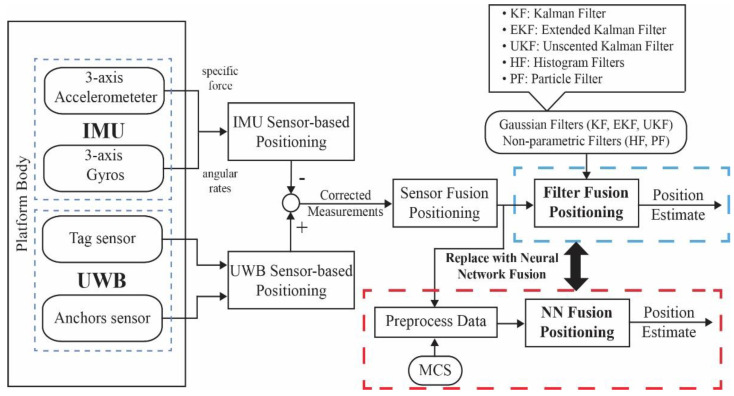
Proposed NN fusion position estimation using the integration of IMU/UWB with MCS data fusion.

Indeed, improving the accuracy of IMU-based systems has been investigated based on their combined use with other sensors or technology. One method involves combining an IMU with magnetometers [38,39]; however, it is not widely used because large magnetic fluctuations exist in indoor environments that affect measurements, although better calibration techniques are being developed [40]. Furthermore, more complicated sensors, including light detection and ranging (LIDAR), have been utilized to provide heading corrections, which can effectively reduce errors [41]. Other common techniques include hyper-algorithms based on suitable sensor fusion, which have been found to be the most effective solutions for positioning systems to achieve sufficient accuracy. Such fusion techniques using IMU and TOF measurements from UWB systems have the advantage of accurately deriving useful information related to the position compared to other methods because they combine the features of different mechanisms to enhance performance [18,42]. This is where the fused UWB system and IMUs can assist in eliminating noise and enhancing the measurements. However, when considering a large range of different dynamic and static rotational and translational motions, the attainable accuracy is limited by the need for situation-dependent adjustment of the accelerometer and gyroscope fusion weights [19]. Hence, we introduce a fusion technique using an ANN with fusion data of IMU/UWB with MCS to increase position accuracy and overcome the aforementioned issues. Figure 2 illustrates the proposed sensor fusion workflow for position estimation. In many applications, IMU-based UWBs are employed to estimate the positions of indoor environments in real time. Commonly used sensor fusion filters may be outperformed, but still suffer from noise and outlier measurements, as previously discussed. Therefore, a fusion technique using an ANN has been proposed and replaced with traditional fusion filters.

Currently, several UWB radio sensor-based devices, including DWM1000 from DecaWave [43] and Pozyx from Pozyx NV [44], have become commercially available and have been deployed in the field of indoor positioning systems, providing centimeter-level accuracy. The Ubisense [45] and Pozyx systems are two well-known positioning systems in which a user carries tags that transmit UWB signals to fixed sensors (anchors) that use the signals to determine the position of the user using the TOF method. In this study, we used the Pozyx system to implement a system design to realize real-time indoor tracking and distancing, which is introduced in the next section.

## 3. Materials and Methods

This section demonstrates the consideration of the measurement system, including the conditioning and calibration of the Tags/IMU sensors, UWB system, MCS, and the mobile robot. Moreover, it describes the ANN model and parameters of the training algorithm to be implemented for fusion positioning.

### 3.1. Measurement System Consideration and Calibration

**Figure 3:**
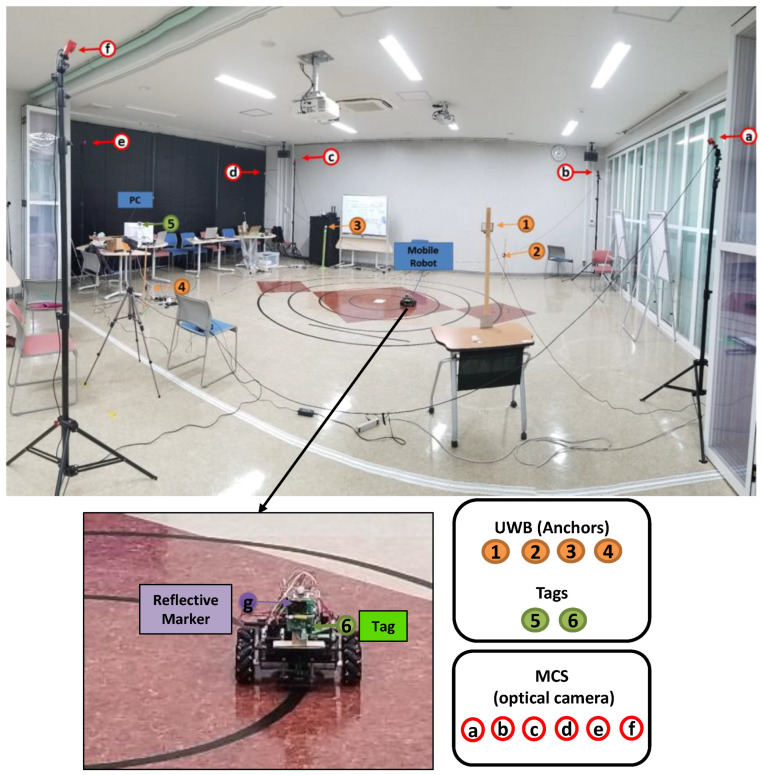
Position measurement system of IMU/UWB and MCS using a mobile robot.

The calibration process is performed in real-time in an LOS scenario in which outliers in the measurement and the communication delay of IMU/UWB sensors cause errors in the estimated position. To ensure the high accuracy of the measurement system and optimal performance of the location estimates, the calibrations of positioning IMU/UWB and OptiT-MCS data have been utilized in dynamic movement with a diverse set of motion recordings using a mobile robot. Figure 3 shows the experimental setup of the measurement system. The IMU/UWB system was established using four anchors (1, 2, 3, and 4) which were distributed at different known positions and two tags/IMU. The anchors are Pozyx devices that work as reference points for the tags and their positioning algorithm. It captures signals, processes data, and provides robust UWB positioning. The UWB configuration parameters that may affect system performance are listed in Table 1. The coordinates of the anchors used are listed in Table 2 and were placed at different heights and in LOS of the tags. Moreover, it was also spread around the tags to increase the chance of receiving a good signal and to cover all directions within the test area.

Similarly, in OptiT-MCS, six optical cameras (a, b, c, d, e, and f) were distributed at different known positions to detect the reflective marker position (g). One Tag/IMU (6) and one retroreflective marker (g) were placed on the mobile robot using a wood holder for positioning data. One more Tag/IMU (5) was used as a master tag, which connected to the PC to collect positioning data from Tag (6) and to communicate with the anchors. Simultaneously, real-time 3D position data were collected from both IMU/UWB and OptiT-MCS-based robot movements at a constant speed. The sampling rates were 11 Hz for UWB and 50 Hz for OptiT-MCS; later, the data measurements were synchronized.

**Figure 4:**
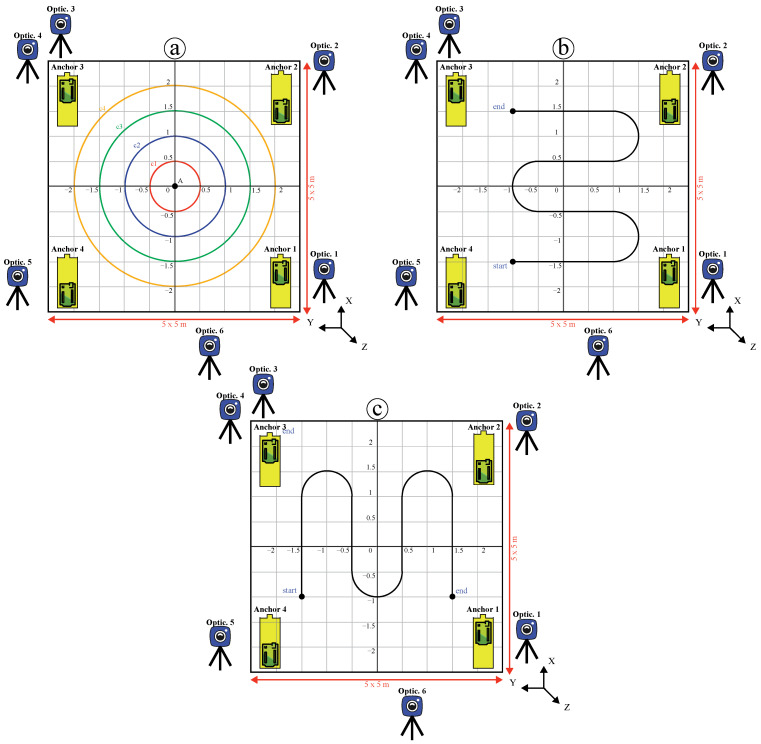
Test area used in three scenarios: (**a**) circular motion, (**b**) random motion on Y-axis, and (**c**) random motion on X-axis.

The proposed system was validated in three experiments with a diverse set of motion recordings inside the testing area (5 m × 5 m) to ensure the generalization of recording data with a high coverage of the proposed test area. One experiment imitated a circular movement, and the second and third represented random movements on the Y-axis and X-axis, respectively, as indicated in Figure 4. The positioning calibration of shape (a) in Experiment 1 was repeated using a mobile robot with different diameters of 0.5, 1.0, 1.5, and 2 m. Thus, four circles with 30 trials each were executed, with 120 trials of circular data recording motion. Subsequently, shapes (b) and (c) were implemented with 15 trials each, totaling 30 trials of random data recording motion. In total, 150 trials of positioning data were collected from both the IMU/UWB and OptiT-MCS.

### 3.2. IMU-UWB-MCS Mobile Robot System Model

In this study, we present an indoor positioning algorithm that fuses IMUs with TOF measurements from UWB and integrates it with MCS data using ANN fusion to guarantee the positioning accuracy. The IMU and UWB transmitter were assumed to be rigidly attached to each other so that the IMU/UWB setup system consists of a network of asynchronous and stationary (rigidly fixed, mounted) transmitters and receivers. Accordingly, we used a set of Pozyx devices, “Creator system” [44], a low-cost hardware that integrates a UWB transceiver in addition to several inertial sensors that provide robust and accurate positioning. Using at least four anchors, the tag can compute its 3D position once it ranges with each anchor position by means of trilateration using the two-way ranging (TWR) protocol. The UWB signals used in Pozyx have a bandwidth of 500 MHz, resulting in 0.16 ns wide pulses in which the system is unaffected by the existence of other communication devices or external noise owing to its high bandwidth and signal modulation. Therefore, it is possible to distinguish several reflections of the signal and perform accurate ranging, even in places with many reflectors.

The developed algorithm was based on real-time measurements obtained from Pozyx devices to assess the approach in a realistic scenario. From such a set of row sensor measurements, measurements from the sensing components of the IMU, such as the accelerometer, gyroscope, and TOF measurements, were directly used for sensor fusion, instead of filtered output quantities, such as position or acceleration. Hence, nothing was disregarded and the maximal advantage was taken of the available information instead of pre-processing measurements, which typically results in the loss of information.

OptiT-MCS was used for performance evaluation, target reference, and comparison with IMU/UWB data fusion using a NN. OptiTrack is a real-time tracking system with a motive license software platform designed to control motion capture systems for various tracking applications. It provides access to camera images, centroids, reconstructed 3D points, and rigid body 6-degrees-of-freedom (DoF) position and orientation with millimeter accuracy. Six optical cameras were placed around the capture volume so that the markers in the volume were visible by at least two cameras at all times. Subsequently, the camera setting configuration was set by Motive such that the marker reflections could be clearly captured and distinguished in the 2D view of each camera. In this study, for the best tracking results, we prepared and cleaned the capture environment before setting up the system by removing unnecessary objects that could block the camera views, covering open windows, and minimizing sunlight. In addition, all infra-red (IR) emitting devices and reflective surfaces within the capture volume were removed or covered prior to calibration.

**Figure 5:**
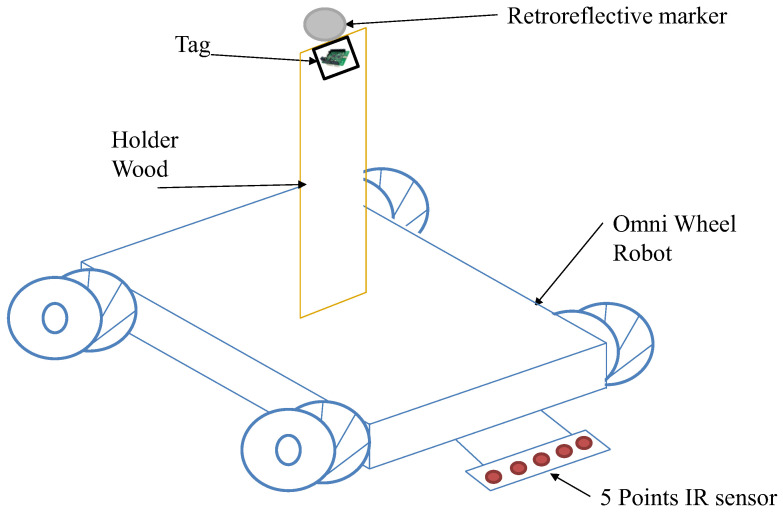
Omni wheels mobile robot attached with Tag/IMU and passive retroreflective marker.

Figure 5 shows a mobile robot attached with Tag/IMU and passive retroreflective marker-based MCS. The Omni wheels mobile robot OSOYOO (model ZZ012318MC) was used to execute the motion at a constant speed to ensure high reliability and robustness of a diverse set of motion recordings in which high accuracy of position data was obtained. The OSOYOO Mega2560 board is fully compatible with the Arduino UNO/Mega2560 that the proposed program was developed with, and the robot automatically moves along the black track line by reading data from a 5-point tracking sensor module. Two trackable sensors were directly placed on the robot using a wooden holder at the same point as the fixed height (z = 15 cm), as indicated in Figure 5. A Tag/IMU-based UWB system and passive retroreflective marker-based OptiT-MCS were used for position tracking. The robot started moving at a constant speed of 160.74 mm/s via remote control by Bluetooth while simultaneously recording positioning data.

Before data calibration, the mobile robot was placed inside the OptiTrack capture volume and the IR emissions emitted by the robot were masked. Consequently, three marker wands (CW-500 calibration wand) were used to calibrate the OptiT-MCS, resulting in the maximum possible accuracy level (a sub-millimeter precision level). Subsequently, the ground plane was set (Motive 1.7 L-Frame) with three markers, and immediately afterwards, a trackable rigid body was created at the origin of the OptiTrack coordinate frame, and the OptiT-MCS was ready for collecting positioning data. This trackable body was physically matched to the UWB origin coordinate frame and used to obtain the position vector of the reflected marker placed on the mobile robot as well as to validate the position differences between the two coordinate frames (UWB and MCS). Accordingly, the information was used to create row position measurement data relating the coordinate frames of the UWB and OptiTrack, which were later fused using the proposed NN model.

### 3.3. Data Collection

The OptiTrack passive retroreflective marker consists of six cameras used for marker position tracking and two OptiTrack OptiHubs controlling hardware modules for handling communication, synchronization, and control of data flow between cameras and computers. The marker cluster used in this experiment was created using a marker 5/16” in diameter positioned to track the movement of the mobile robot. The robot 3D position-based-marker was recorded in meters at a sampling rate of 50 Hz. In contrast, the UWB with one Tag/IMU was utilized to track the position of the mobile robot from the same coordinate point of the retroreflective marker; however, the position was recorded in millimeters at a sampling rate of 11 Hz. Therefore, positioning data were simultaneously acquired using an OptiT-MCS and UWB system at different sampling rates. To compare the trajectories captured by the two systems running at different sampling rates, a custom MATLAB code was developed to down-sample both the 50 Hz OptiT-MCS data and 11 Hz UWB data into a 10 Hz dataset. The records between systems have the potential to be mismatched by a few milliseconds because independent systems software was used with different sampling rates for both systems, as well as various constraints on the CPU scheduling control that operates in a Windows 10 non-real-time operating system environment, even though they should have identical timestamps. Accordingly, for synchronization and matching the result to the same start and end data row measurement, normalization and cross-correlation were developed using MATLAB. Both the OptiT-MCS and UWB systems were connected to a computer via USB 2.0.

### 3.4. Artificial Neural Network Model

**Figure 6:**
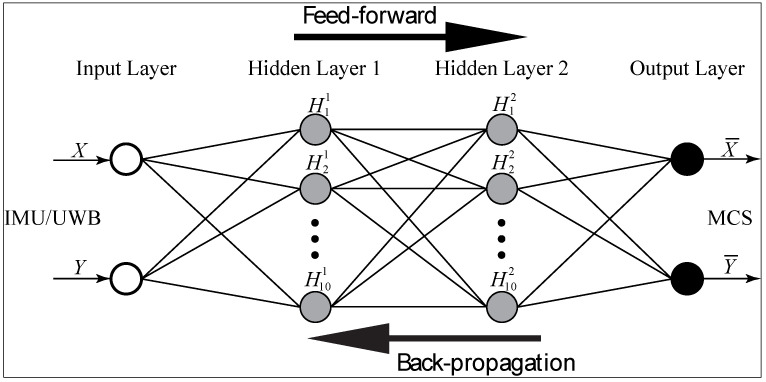
Schematic of four-layer artificial neural network fusion data.

With the collected data (150 experimental trials) from IMU/UWB and MCS, an ANN fusion was built using the Neural Network Toolbox of MATLAB. Figure 6 illustrates a four-layer neural network implemented based on the Levenberg–Marquardt backpropagation (LMBP) method [46]. The input layer receives the 2 row position values (X and Y) of the IMU/UWB sensor, while each hidden layer has 10 neurons (H1-H10) with a tangent sigmoidal hyperbolic as the activation function as shown in Equation (1). The output layer has two neurons with a linear activation function as shown in Equation (2) [47]. The outputs ($\bar{X}$ and $\bar{Y}$) are row position values of the MCS sensor that represents the target position values of the input data. A trial and error approach was used to determine the parameters of the ANN (number of neurons in the hidden layer and activation functions) after multiple tests [48]. In this research method, the design of the network architecture was determined to achieve the simplest ANN structure along with the least output error. The input and output data were normalized using the Min–Max [49] method in MATLAB, where the acquired data were randomly divided into the following three sets to avoid any bias: training 70%, validation 15%, and testing 15%.

Table 3 lists the specifications and values of the parameters used in the LMBP-ANN model, as well as the development environment used for learning the prediction model. The training and validation sets were utilized to develop model structures, determine weights and biases, and to avoid overfitting by validating an optimal parameter set. The testing set was only used to explore the performance of the trained model and to confirm its generalizability.

(1) $$y_{j}=\frac{e^{x_{j}}-e^{-x_{j}}}{e^{x_{j}}+e^{-x_{j}}}$$

(2) $$y_{i}=X_{j},X_{j}=\sum_{i=1}^{m}w_{ij}y_{i}+b_{j}$$

where m denotes the number of neurones in the output layer, $w_{ij}$ denotes the weight of connections between layers *i* and *j*, $y_{i}$ indicates the output of neurones in layer *i*, and $b_{i}$ corresponds to the bias of neurones in layer *j*.

## 4. Results and Discussion

In this section, we present the results of the proposed method. Accordingly, the analysis and experimental results for the 2D position measurement system using the IMU/UWB and MCS are presented. Subsequently, the training and evaluation results of the proposed NN fusion model were implemented. Ultimately, the performance and position accuracy of NN fusion were validated.

### 4.1. Calibration Position Data

A series of experiments were performed to verify the position estimation accuracy of the proposed NN fusion model. A diverse set of motion recording data-based IMU/UWB and OptiT-MCS systems using a mobile robot was collected. However, as previously mentioned, the frequency of measurements from the IMU/UWB system (11 Hz) was significantly lower than the OptiT-MCS data rate (50 Hz), as indicated in the before pre-processing row of Table 4. Therefore, the fusion of both systems was used to combine their complementary features. Hence, for synchronization and matching the result to the same start and end data row measurement, normalization and cross-correlation were developed using MATLAB code, and the results are shown in Table 4 in row data after pre-processing using a 10 Hz sampling rate for all data sets. Subsequently, all these data were combined using the NN fusion model, which was implemented in MATLAB to obtain a graphical representation of the resulting measurements. It can be observed that 83,508 calibrated data points were fed into an NN fusion model to estimate the position with a high level of accuracy.

**Figure 7:**
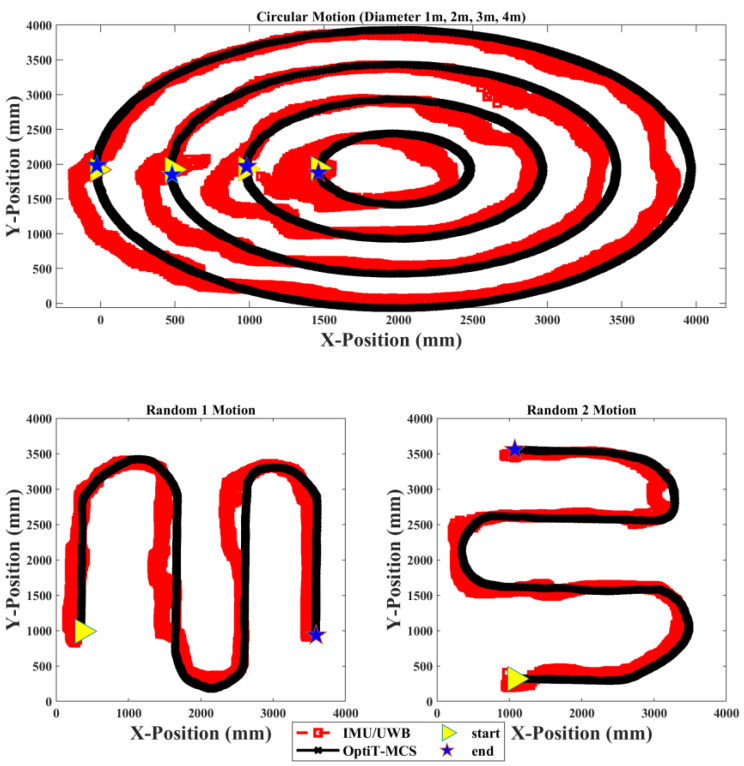
Trajectory position tracking of the mobile robot in X-Y coordinates by IMU/UWB system compared to OptiT-MCS for 150 trials of three types of experiments.

Figure 7 illustrates the trajectory position estimated using the mobile robot in the X-Y coordinates by IMU/UWB and OptiT-MCS for 150 trials in three types of experiments. The right-pointing triangle represents the start point of the mobile robot tracking, whereas the pentagram represents the end point of the path or destination points. The results of calibration indicate that the positional error of the IMU/UWB deviated along the path, and it was impossible to obtain an accurate measurement of the initial start point (right-pointing triangle) under an increasing error owing to the processing duration and USB communication delay between both tags and anchor devices, which is not instantaneous. Furthermore, the mobile robot was carefully controlled to move in a constant speed with low vibration to ensure the high reliability and robustness of a diverse set of motion recordings, but the rigid drives and joints negatively affect the measured data along the path. Thus, the trajectory obtained from the IMU/UWB system presented an average error of 24.2 cm with regard to the OptiT-MCS (millimeter average error) path for all data measurements. In addition, when a large range of different dynamic and static rotational and translational motions is considered, as in our implemented experiments, the attainable position accuracy is limited and the NN fusion model is required to overcome this issue.

### 4.2. Neural Network Fusion

In the proposed NN fusion model, the calibrated position data flow from the input to the output layers through hidden layers. Then, the backpropagation method was used to minimize the sum of squares error signals of the output by adjusting the coefficients of synapse weight until it reached the highest performance with minimum MSE. Moreover, the generation of a dataset is critical for training the algorithm. Therefore, position data were collected from different types of motions with 150 experimental trials using a mobile robot and covering the space of the testing area (5 m × 5 m). A 2 × 83,508 matrix of measurement position data points from the IMU/UWB system was fed into the NN fusion algorithm. Similarly, during the calibration process, the target output of the training algorithm was the position of a 2 × 83,508 matrix obtained from the reference system, namely the OptiT-MCS. One row represented the X position, whereas the second row represented the Y position for both systems.

The performance and validation of the proposed NN fusion model primarily depend on its ability to predict the experimental output data with reasonable accuracy. All evaluation processes using data of the IMU/UWB and OptiT-MCS systems in the NN fusion model exhibited coefficient of correlation R values higher than 0.99 compared with 0.98 in IMU/UWB system due to outliers in the measurements. The correlation between the observed and simulated position values was determined for the training, validation, and test data with high performance, that is, 0.99501, 0.99506, and 0.99507, respectively. Moreover, outliers in the IMU/UWB measurements were solved using the proposed NN model as the performance R increased by 1.25%.

**Figure 8:**
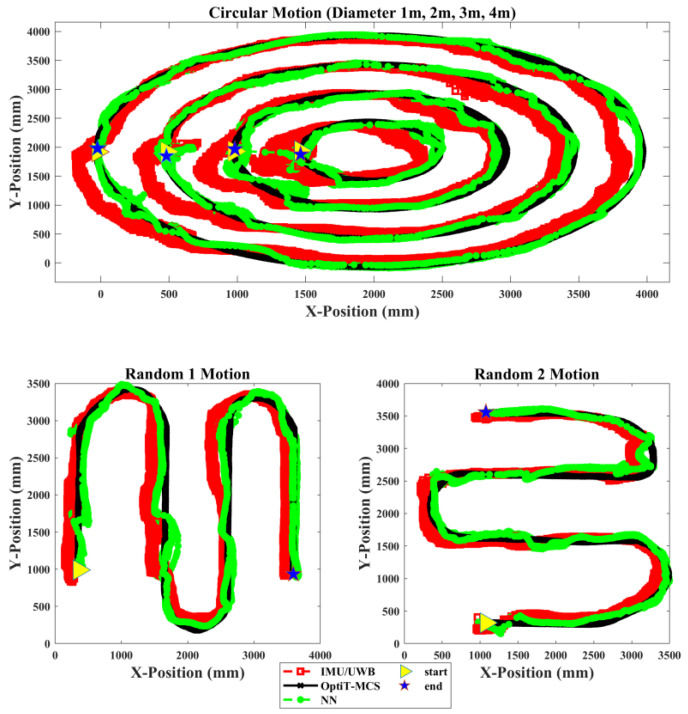
Trajectory position tracking of mobile robot in X-Y coordinates by IMU/UWB system and proposed NN compared to OptiT-MCS for one trial of three types of experiments.

The performance and effectiveness of the proposed NN fusion model were further evaluated using a new independent input position dataset on which the model had never been trained. Accordingly, two new vectors, with X and Y input position data, were fed to the NN model to predict the position of the mobile robot that moved along the different types of path-based motions. Figure 8 illustrates the trajectory position tracking of the mobile robot in the X-Y coordinates by the IMU/UWB system compared to the proposed NN model relative to OptiT-MCS for one trial of the three types of experiments. Evidently, the proposed NN fusion model successfully predicted the position of the mobile robot and successfully tracked the target position (OptiT-MCS) with less deviation error than the IMU/UWB system. Moreover, the results also verify that the proposed NN fusion can effectively correct and accurately estimate the position with sufficient performance and the problem of the communication gap in the IMU/UWB system at the initial start point (see right-pointing triangle) because the processing duration and USB communication can be significantly evitable.

### 4.3. System Evaluation

It is challenging to differentiate the results presented in Figure 7 and Figure 8. Thus, several statistical methods were required to evaluate the results in a single quantifiable comparative analysis. Accordingly, the accuracy and performance of the proposed model were evaluated using the following three different metric errors: the mean absolute deviation (MAD), mean absolute percentage error (MAPE), and root mean square error (RMSE), as shown in Equations (3)–(5):

- MAD: This was calculated by measuring the sum of the absolute differences between the actual value ($A_{t}$) determined by the OptiT-MCS and the forecast ($F_{t}$) positions provided by the IMU/UWB and NN systems divided by the number of observations.

(3) $$\mathrm{MAD}=\frac{\sum_{t=1}^{n}\left|A_{t}-F_{t}\right|}{n}$$

- MAPE: This was calculated by expressing the average of absolute errors divided by actual observation values as a percentage of the total positions of the mobile robot travelling from the first point to the end of each experiment.

(4) $$\mathrm{MAPE}=\frac{\sum_{t=1}^{n}\frac{\left|A_{t}-F_{t}\right|}{A_{t}}}{n}\times 100$$

- RMSE: This was calculated as the square root of the sum of squared errors divided by the number of observations.

(5) $$\mathrm{RMSE}=\sqrt{\frac{\sum_{t=1}^{n}{\left(A_{t}-F_{t}\right)}^{2}}{n}}$$

Table 5 presents the MAD, MAPE, and RMSE results in position with the proposed fusion NN model and the IMU/UWB system. Overall, the results indicate that the MAPE error in the position estimation was improved by 17.56% and 7.48% in the X and Y coordinates, respectively, in the case of the proposed fusion NN model. Moreover, multiple results were obtained for evaluating the performance and accuracy of IMU/UWB and the proposed fusion NN model with different tracking motion scenarios. The experimental results reveal that the proposed NN with circular motion in the X and Y coordinates performed well compared to other random motion measurements in terms of RMSE. The best outcome for the NN model was where circular motion was in the X coordinate, as a result of the 74.93 mm prediction accuracy in terms of RMSE as the error decreased from 56.88% to 10.96% in the MAPE metric. In contrast, the worst tracking motion scenario was in Random 1 motion as a result of the 185.15 mm prediction accuracy in the Y coordinate in terms of RMSE. Furthermore, as indicated in the table below, for both measurement methods, the influence of the experiment type in error on the position estimation accuracy increased with random motion compared to circular motion, as the mobile robot autonomously followed its path in a dynamically changing environment with a longer distance than circular motion. This holds for all data sequences, and the error type is an expected behavior.

**Figure 9:**
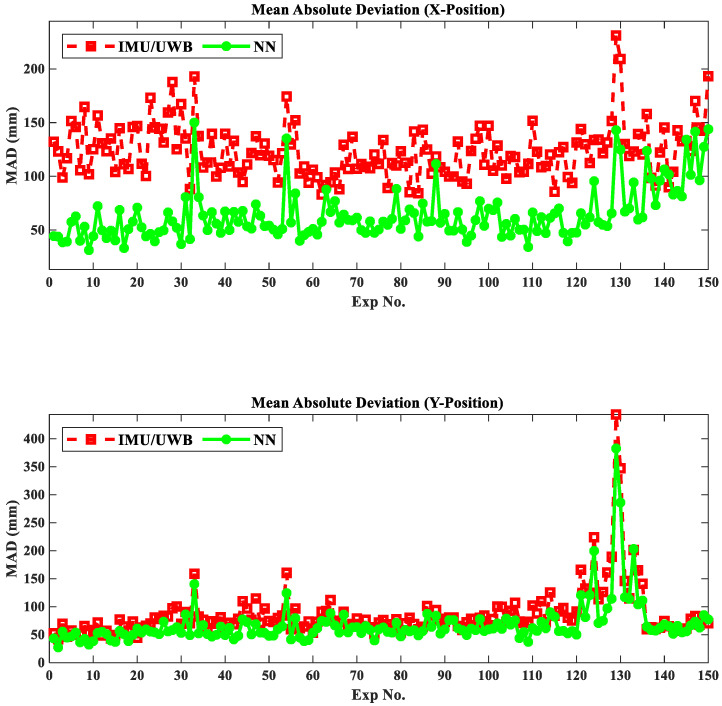
Metric errors prediction results using MAD in X and Y coordinates of 150 experimental trials.

For a visual representation of the position accuracy during the entire experimental process, Figure 9 depicts the position estimation accuracy in millimeters in terms of MAD for 150 experimental trials. From this figure, it can be observed that there is a significant enhancement in the prediction accuracy of the proposed fusion NN model compared to the IMU/UWB system, which is 79.71 mm for the best case in the X coordinate and 89.97 mm for the worst case in the Y coordinate. Furthermore, the proposed fusion NN model performed better in the X-and Y-coordinates in circular motion (Exp. 1~120) compared to random motion (Exp. 121~150) and better than 40.77% and 16.94% positioning accuracy improvements in circular and random motions, respectively, over the IMU/UWB system. Ultimately, the positioning accuracy improvement can be further realized in either the X or Y coordinates by both IMU/UWB and NN systems in Random 2 motion (Exp. 136~150) compared with Random 1 motion (Exp. 121~135).

## 5. Conclusions

The precise localization of human operators in robotic workplaces in indoor environments is a critical requirement that must be satisfied to develop human–robot interaction tasks. Tracking the movements of human and robot positions and dynamically estimating the mutual distances with high accuracy as our proposed method performed will definitely contribute to a stronger interaction between human and robot. Positioning is one of the most vital and challenging phases in indoor tracking systems. Accordingly, different technologies have been developed to enhance position performance. In real-time processes, there is variability and uncertainty in that, in some situations, positioning cannot be evaluated using traditional mathematical models. Furthermore, while IMU/UWB fusion technology provides high accuracy positioning besides many other features, it still suffers from limited accuracy, particularly when a large range of different dynamic and static rotational and translational motions are considered. In addition, a communication gap between the tags and anchor devices at the initial connection was detected. Therefore, in this study, a novel position estimation approach using NN fusion is developed to guarantee the positioning accuracy of moving robots by integrating IMU/UWB with OptiT-MCS data fusion.

The proposed approach was experimentally tested and verified in accurate position estimates when compared to data from the IMU/UWB system relative to the OptiT-MCS reference system, giving an enhancement average MAPE of 17.56% and 7.48% in the X and Y coordinates, respectively, and the coefficient of correlation R value greater than 99%. These results, using databases containing 83,508 position data from three different motion experiments, highlight the ability to generalize the designed networks. Likewise, the results reveal that the proposed NN fusion is capable of maintaining high accuracy in position estimates and prevents drift errors from increasing unboundedly. In future work, complex interaction tasks that consider the spatial relationships between the robot and the human will be developed based on the proposed NN fusion model.

## Figures and Tables

**Table 1:** Configuration parameters of the UWB system.

UWB Setting	Values
Channel	2
Positioning protocols	Time-Of-Flight (TOF)
Update rate	11 Hz
Bandwidth	499.2 MHz
Data bitrate	850 Kb/sec
Pulse repetition frequency (PRF)	64 M Hz
Preamble length	2048
Transmit power Tx gain	15.5 dB

**Table 2:** The anchors’ coordinates of UWB system.

Anchors’ Coordinates			
Anchor No.	X (mm)	Y (mm)	Z (mm)
1	−1000	0	1500
2	4500	0	600
3	4180	4230	1000
4	−1000	4200	400

**Table 3:** The specifications and parameters of ANN model and development environment.

Training Parameters/Component	Values	Note
Neural network model used	Feed forward	
Input nodes	2	X, Y Position of IMU/UWB Sensor
Hidden layer	2	
Hidden layer neurons	20	
Output layer neurons	2	
Output nodes	2	$\bar{X},\;\bar{Y}$ Position of MCS Sensor
Training network algorithm	LMBP	
Training percentage	70	
Testing percentage	15	
Validation percentage	15	
Transfer function hidden layers	Tansig	
Transfer function output layer	Pure line	
Data division	Random	
No. of epochs	1000	
Validation checks (iterations)	6	
Performance	Mean squared error (MSE)	
IDE	MATLAB R2019a	
Operating System	Window 10	
CPU	Intel(R) Core(TM) i9-9900K CPU @ 3.60 GHz	
Memory	64 GB	

**Table 4:** Data point calibrations of tracking position of the three experiments for different algorithms.

Experiment Type /Sampling Rate (Hz)	IMU/UWB	MCS	IMU/UWB	OptiT-MCS	Fusion IMU/UWB + OptiT-MCS
	11	50	10	10	10
	**Before Pre-Processing**		**After Pre-Processing**		
Circular Motion	63,568	294,405	57,772	59,033	58,797
Random 1 Motion	13,253	62,855	12,380	12,589	12,393
Random 2 Motion	13,619	61,202	12,194	12,402	12,318
Total	90,440	418,462	82,346	84,024	83,508

**Table 5:** Metric errors prediction results using MAD, MAPE, and RMSE in X-Y axis of the three experiments for different algorithms.

Experiment Type	IMU/UWB	NN	IMU/UWB	NN
	X Coordinate Error		Y Coordinate Error (mm/%)	
	MAD/MAPE/RMSE (mm/%/mm)		MAD/MAPE/RMSE (mm/%/mm)	
Circular Motion (Exp. 1~120)	120.07/56.88/142.3	57.49/10.96/74.93	76.51/26.34/96.55	58.94/10.78/77.99
Random 1 Motion (Exp. 121~135)	142.08/10.61/166.4	75.34/6.08/98.81	186.39/16.42/228.86	146.44/12.11/185.15
Random 2 Motion (Exp. 136~150)	132.85/9.36/158.59	106.30/7.15/135.7	66.91/8.39/82.11	64.54/5.77/80.60
Total Average error	131.67/25.62/155.76	79.71/8.06/103.14	109.94/17.03/135.84	89.97/9.55/114.58

## Data Availability

The data presented in this study are available on request from the corresponding author. The data are not publicly available because the data may involve confidential information of our research institution.
